# Evolution of orbital angular momentum spectrum of broadband Laguerre–Gaussian beam in OPCPA process

**DOI:** 10.1038/s41598-022-27148-0

**Published:** 2023-01-02

**Authors:** Aotian Wang, Lianghong Yu, Jinfeng Li, Xiaoyan Liang

**Affiliations:** 1 State Key Laboratory of High Field Laser Physics, Shanghai Institute of Optics and Fine Mechanics, Chinese Academy of Sciences, Shanghai, 201800 China; 2 Center of Materials Science and Optoelectronics Engineering, University of Chinese Academy of Sciences, Beijing, 100049 China

**Keywords:** Optics and photonics, Optical physics

## Abstract

In this study, we numerically simulate the evolution of the orbital angular momentum (OAM) spectrum of a vortex laser beam in the optical parametric chirped pulse amplification (OPCPA) process, which is an effective technical method to realize ultra-intense and ultra-short vortex laser amplification. The results show that the proportion of the vortex laser beam with 100% topological charge (TC) of 1 decreases to 97.44% with the enhancement of the saturation amplification after amplification by a 15 mm length LBO pumped by a 526.5 nm laser with a pump intensity of 1.74 GW/cm^2^. Conversely, the beams with other topological charges generate and increase with the amplification. The simulation results are consistent with our previous experimental results. Meanwhile, compared with non-collinear OPCPA, collinear OPCPA can maintain well the proportion of TC $l = 1$.

## Introduction

Since vortex beam was proved by A. Allen et.al in 1992 to carry the orbital angular momentum (OAM)^1^, it has been widely applied in optical communication^2,3^, depleted beam in stimulated-emission-depletion microscopy (STED)^4^, capture particles in optical trapping^5,6^, optical manipulation^5^, etc. The vortex beam has a helical phase, in which the phase varies from 0 to 2$\pi$ *l* surrounding the center of the beam, causing a singularity both in the center of the phase and intensity distributions. In the helical phase of 2$\pi$ *l*, ‘*l*’ is defined as topological charge (TC), which determines the order of the OAM states.

In strong field physics, the vortex beam with an ultra-short pulse duration and ultra-high intensity has been used in particle accelerations^7,8^. Most vortex beam generation methods,such as the transmissive spiral phase plate (TSPP)^9^, liquid crystal spatial light modulator (LC-SLM)^10^, and computer generated holography^10,11^, are restricted by the low damage threshold of devices in these methods, which has limited their application in the generation of strong field vortex beams. In recent years, there have been solutions in the generation of strong field vortex beams, such as the reflective spiral phase plate^7^, backward stimulated Raman scattering^12^, and plasma hologram^13^.

OPCPA is a significant technical route for achieving a higher peak power vortex beam, which has been used in both of near infrared^14^ and mid infrared bands^15^ vortex laser amplification. In our previous experimental work, a broadband vortex beam with a spectrum of 760–830 nm was amplified from 0.5 to 40 mJ by an lithium triborate (LBO)—OPCPA with a non-collinear structure, and 1 TW was then generated with a compressed duration of 30 fs^14,16^. However, we observed that the proportion of TC with decreased in the OPCPA process from 72.7 to 67.51%, whereas the proportion remained almost unchanged in the compression process.

OAM spectrum is essential for evaluating the quality of conventional vortex beam. OAM spectrum represents the intensity weights of TCs in single beam^17^. The OAM spectrum would be reflected on spot and phase of vortex beams, which means OAM spectrum has important influence on the applications of vortex beams. For broadband vortex beams in the experiments, their OAM spectrum at different wavelengths are diverse owing to the limit of the generation devices. For example, if the TSPP is designed to generate vortex beams of $l=1$, the existence of the TSPP refractive index change with the wavelength causes the $l=1$ proportion of the OAM spectrum to not be equal to 100% at different wavelengths except the designed wavelength of the TSPP. The modulation of the OAM spectrum at different wavelengths, is referred to as the ‘topological charge dispersion’^18^.

In this work, we numerically simulated the evolution of the orbital angular momentum (OAM) spectrum of the vortex laser beam in the OPCPA process. The results show that the proportion of the Laguerre–Gaussian (LG) signal beam with a 100% topological charge (TC) of 1 before amplification decreases with the enhancement of the saturation amplification to 97.44% after amplification by a 15 mm length LBO pumped by a 526.5 nm laser with a pump intensity of 1.74 GW/cm^2^. With the enhancement of the amplification saturation effect, the distortion of the spatial near-field distribution and phase distribution of the amplified pulse is the main reason for the change of the OAM spectrum. We analyzed the OAM spectrum with different wavelengths in our work. Compared with the non-collinear OPCPA (NOPCPA) structure, the orbital angular momentum spectrum of the collinear OPCPA (COPCPA) structure remains better before and after amplification.

## Numerical model

The input signal beam is an LG01 beam, and the electric field expression is given by^1^

1 $$ E\left( {r,\varphi ,z} \right) = \frac{C}{{\omega _{0} }}\left( {\frac{{\sqrt 2 r}}{{\omega \left( z \right)}}} \right)^{l} L_{p}^{l} \left( {\frac{{2r^{2} }}{{\omega \left( z \right)^{2} }}} \right)\exp \left( { - \frac{{r^{2} }}{{\omega \left( z \right)^{2} }}} \right)\exp (il\varphi )\exp \left( {i(2p + l + 1)\arctan \left( {\frac{z}{{z_{R} }}} \right)} \right)\exp \left( {\frac{{ - ikr^{2} z}}{{2(z^{2}  + z_{R}^{2} )}}} \right) $$

where *C* is a constant, $\omega _0$ is the radius of the LG00 beam waist, *z* is the propagation distance, $z_R$ is the Rayleigh range ($z_R=\pi \omega _0^2/\lambda$), $\omega {(z)}$ is the beam radius ($\left( {\omega (z) = \omega _{0} \sqrt {1 + (z/z_{R} )^{2} } } \right)$), $L^l_p$ the Laguerre polynomial, *l* is the topological charge ($l=1$), *p* is the radial mode indices ($p=0$), and $k=2\pi /\lambda$ is the wave vector.

The amplified vortex beam can be obtained by numerically solving the coupled wave equations (2) with the split-step Fourier method^19^.

2 $$\begin{aligned} \begin{aligned} \frac{{\partial {{{\tilde{E}}}_s}}}{{\partial z}} = - i\left( {\frac{{{\omega _s}}}{{{n_s}c}}} \right) {d_{eff}}{{\tilde{E}}_p}{{{\tilde{E}}}_i}*\exp ( - i\Delta kz) \\ \frac{{\partial {{{\tilde{E}}}_i}}}{{\partial z}} = - i\left( {\frac{{{\omega _i}}}{{{n_i}c}}} \right) {d_{eff}}{{{\tilde{E}}}_p}{{{\tilde{E}}}_s}*\exp ( - i\Delta kz) \\ \frac{{\partial {{{\tilde{E}}}_p}}}{{\partial z}} = - i\left( {\frac{{{\omega _p}}}{{{n_p}c}}} \right) {d_{eff}}{{{\tilde{E}}}_s}{{{\tilde{E}}}_i}*\exp (i\Delta kz) \\ \end{aligned} \end{aligned}$$

where subscripts *p*, *s*, and *i* represent the pump, signal and idle beam, respectively. ${{\tilde{E}}}_p$,${{\tilde{E}}}_s$, and ${\tilde{E}}_i$ are the spatial complex electric field of the pump, signal and idle beam, respectively. It should be noted that their temporal electric field expressions do not have a time phase in our simulation with a duration of the ns level. According to [17,19], to calculate the OAM spectrum, the beam spatial electric field expression should be expanded through helical harmonics $\exp {(il\varphi )}$ as

3 $$\begin{aligned} E\left( {r,\varphi ,z} \right) = \frac{1}{{\sqrt{2\pi } }}\sum \limits _{l = - \infty }^\infty {{a_l}(r,z)\exp (il\varphi )} \end{aligned}$$

where

4 $$\begin{aligned} {a_l}\left( {r,z} \right) = \frac{1}{{\sqrt{2\pi } }}\int _0^{ + \infty } {E\left( {r,\varphi ,z} \right) } \exp ( - il\varphi )d\varphi \end{aligned}$$

Then, the coefficient of the *l*- th order helical harmonic is as follows

5 $$\begin{aligned} {C_l} = \int _0^{ + \infty } {{{\left| {{a_l}\left( {r,z} \right) } \right| }^2}rdr} \end{aligned}$$

The proportion of the *l*-th order helical harmonic (or the TC *l*) is

6 $$\begin{aligned} PP(l) = \frac{{{C_l}}}{{\sum \limits _{q = - \infty }^{ + \infty } {{C_q}} }} \end{aligned}$$

For broadband signal beam, the expression of the total OAM spectrum is as follows

7 $$\begin{aligned} P{P_{total}}(l) = \int \limits _{\lambda = 760\mathrm{{ nm}}}^{\lambda = 840\mathrm{{ nm}}} {PP(l,\lambda ){I_{spectrum}}(\lambda )} d\lambda \end{aligned}$$

where is the OAM spectrum with a different wavelength, $PP_{total}$ is the total OAM spectrum with the full bandwidth, and $I_{spectrum}$ is the normalized intensity of the spectrum ($\int {{I_{spectrum}}(\lambda )d\lambda } = 1$).

## Results and discussion

**Figure 1:**
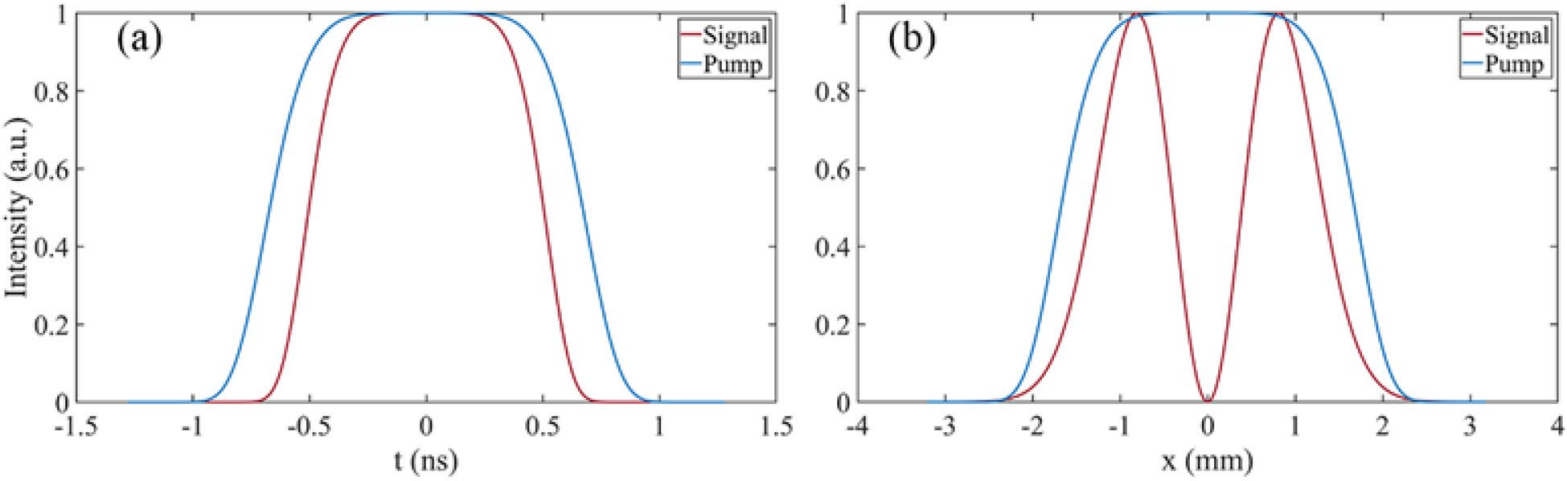
(**a**) Temporal profile and (**b**) spatial beam profile of input signal (red line) and pump (blue line) beam.

The TC of the input signal beam is $$l=1$$ with a spectrum of 760–840 nm. The injected signal energy is 2 mJ with a duration of 1.2 ns and a spot radius of 1.2 mm. The wavelength of the pump beam is 526.5 nm with a duration of 1.6 ns and the spot radius is 2 mm. The temporal waveforms and spatial profiles of injected beams are shown in Fig. 1a, b, respectively. The temporal waveform of signal and pump beam were set as three order super-Gaussian function. The spatial profile of pump beam was set as three order super-Gaussian beam and the spatial profile of signal beam was set as LG01 beam represented by the red curves in Fig. 1b, in which the depression on the curve represents the singularity in the vortex beam space. The maximum pump intensity was $$I_{pump} = $$1.74 GW/cm$$^2$$. The LBO was used as the amplifier with a phase match angle of $$\theta =90^\circ $$, $$\Phi =13.85^\circ $$ and length of 15 mm.

**Figure 2:**
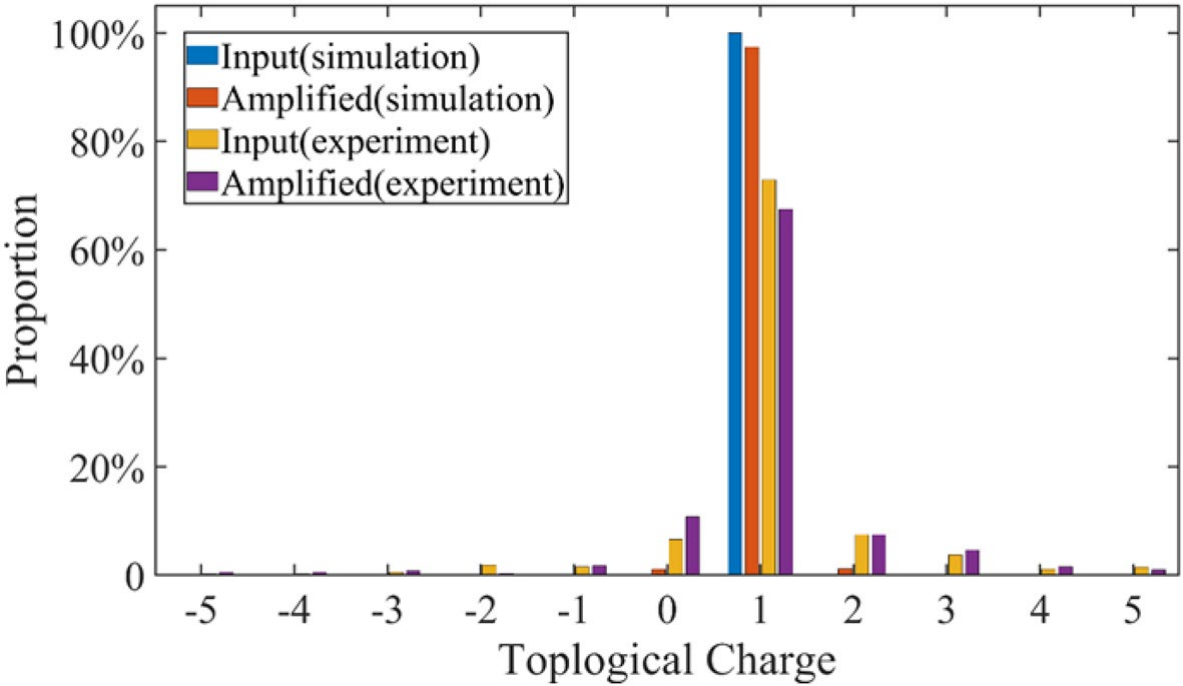
Simulated OAM spectrum of input broadband signal pulse (blue), simulated OAM spectrum of amplified pulse (orange), experimental input OAM spectrum (yellow), and experimental OAM spectrum after amplification (purple).

We simulated the change of the proportion of different TCs before and after amplification, and then compared them with the results of the experiment in Ref [14]. The pump intensity, injected signal intensity, and parameters of the LBO crystal were similar in the experiment and simulation. As shown in Fig. 2, in our simulated results, the proportion of $l=1$ in the total OAM spectrum of the LG beams decreases from 100 to 97.44% after the optical parametric amplification (OPA) at a crystal length L= 15 mm. Meanwhile, the proportion of $l=0$ increases from 0 to 1.13%, whereas the proportion of $l=2$ increases from 0 to 1.23%. In the experiment, owing to the imperfection of the phase plate, the proportion of the injected laser beam with $l=1$ was 72.87%, rather than 100% under ideal conditions. The proportion of the laser beam with $l=0$ was 6.58% and $l=2$ was 7.45%. After amplification, the proportion of $l=1$ decreased from 72.87 to 67.5% and the proportion of $l=0$ and $l=2$ increased from 6.58 and 7.45 to 10.84% and 7.47%, respectively. It can be observed that the proportion of vortex beam injected with pure $l=1$ decreases, and vortex beam of other TCs generate and the proportions increases. The trend of the change of TCs in our simulated results is consistent with our previous experimental results. In the following sections, we discuss the evolution of the OAM spectrum in non-collinear and collinear OPCPA, respectively.

### Non-collinear OPCPA structure

**Figure 3:**
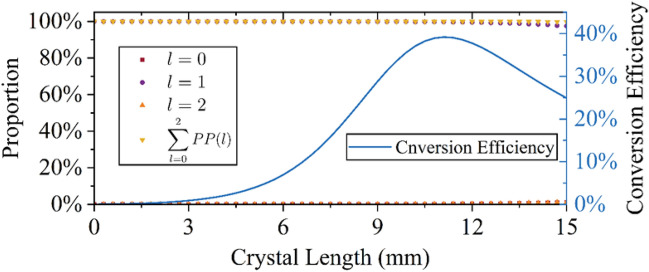
Change of OAM spectrum (left y-axis) and change of conversion efficiency (right y-axis) with crystal length.

To study the evolution of the OAM spectrum of the vortex beam under different amplification conditions of the OPA, including the low gain (low pump intensity), linear gain (medium pump intensity), and saturation gain ( high pump intensity), the maximum pump intensity is 1.74 GW/cm$^2$ which can achieve saturation amplification with a crystal length of 15 mm. The injected signal beam is a broadband laser with 100% TC of $l=1$. When the pump intensity is 1.74 GW/cm$^2$, the relationship between the change of the different TCs and the crystal length is shown in Fig. 3. The conversion efficiency is also shown in Fig. 3, represented by the blue curve. The maximum conversion efficiency occurred when the crystal length was 11.4 mm. With the further increase of the crystal length, saturation amplification occurred.

It can be observed that the proportion of the input TC ( $l=1$) decreases with the increase of the crystal length and decreases to 97.44% at L = 15 mm. Meanwhile the proportions of the other TC ( $l=0,\, l=2$) increase with the crystal length, the proportions of $l=0$ and $l=2$ increase to 1.13% and 1.23% at $L=15$mm, respectively. The longer the crystal length is, the faster $l = 1$ the decrease and $l = 0$ and $l = 2$ increases. Moreover, the sum proportion of the three TCs decreases with the crystal length, which indicates that the other proportion of TCs appears and increases with the crystal length. The increase of the proportions of other TCs can also be observed in Fig. 2.

**Figure 4:**
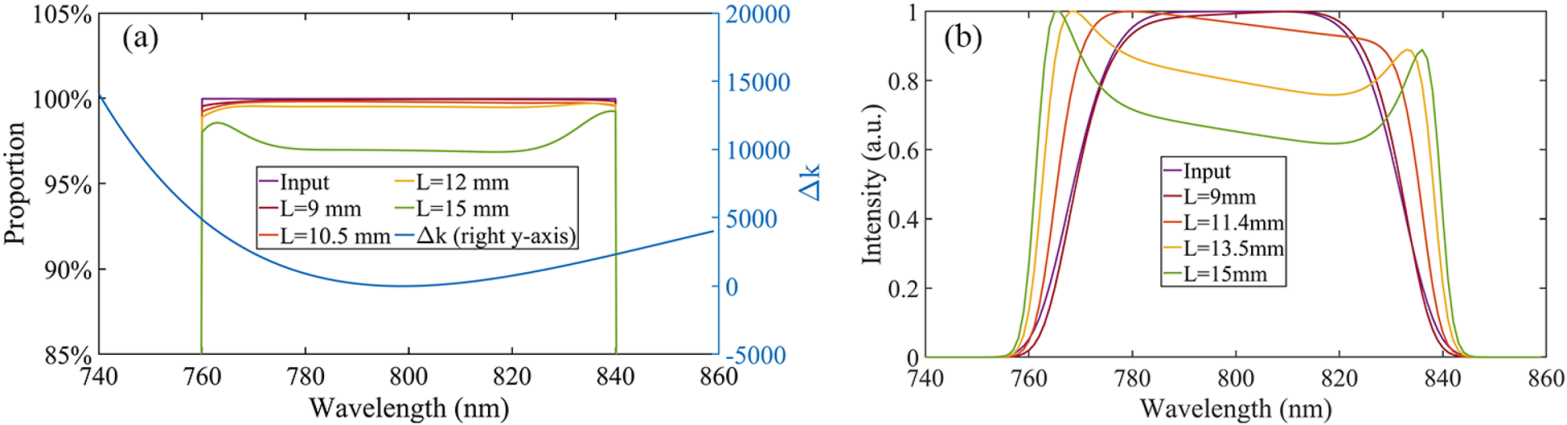
(**a**) OAM spectrums with wavelengths at different crystal lengths (left y-axis) and the value of the wave-vectors mismatch with the wavelength (right y-axis). (**b**) Amplified spectra at different crystal lengths.

For broadband vortex beams, it is more suitable to show the OAM spectrum in different wavelengths. The evolution of the amplified OAM spectrums (only $l=1$) of 760–840 nm with different wavelengths and crystal lengths are shown in Fig. 4a. It can be observed that the proportions of $l=1$ for the full spectrum decrease with the increase in the crystal length. When the saturation effect of the amplification is not significant (L $\le$ 12 mm), the proportion of the $l=1$ laser is nearly independent of the wavelength in the spectral region, which is represented by the blue line shown in Fig. 4a. However, the proportion of $l=1$ of the two wing parts of the spectrum increases slightly when the saturation is significant at a crystal length of 15 mm. The protruding parts on the OAM spectrum can matchs with those on the amplified spectrum, which are shown in Fig. 4b, indicating that the evolution of proportion of $l=1$ is closely related to the degree of amplification saturation.

**Figure 5:**
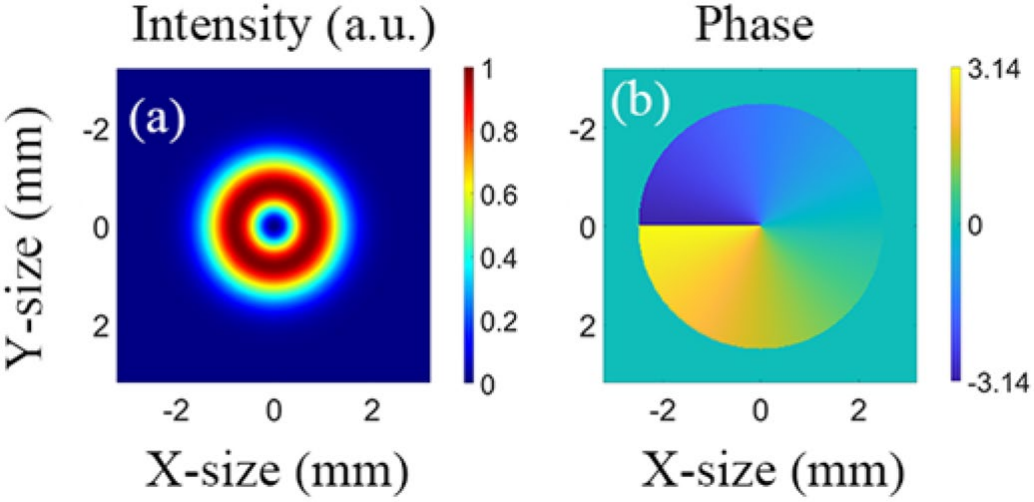
(**a**) Intensity distribution and phase distribution (**b**) of input ideal LG01 beam.

**Figure 6:**
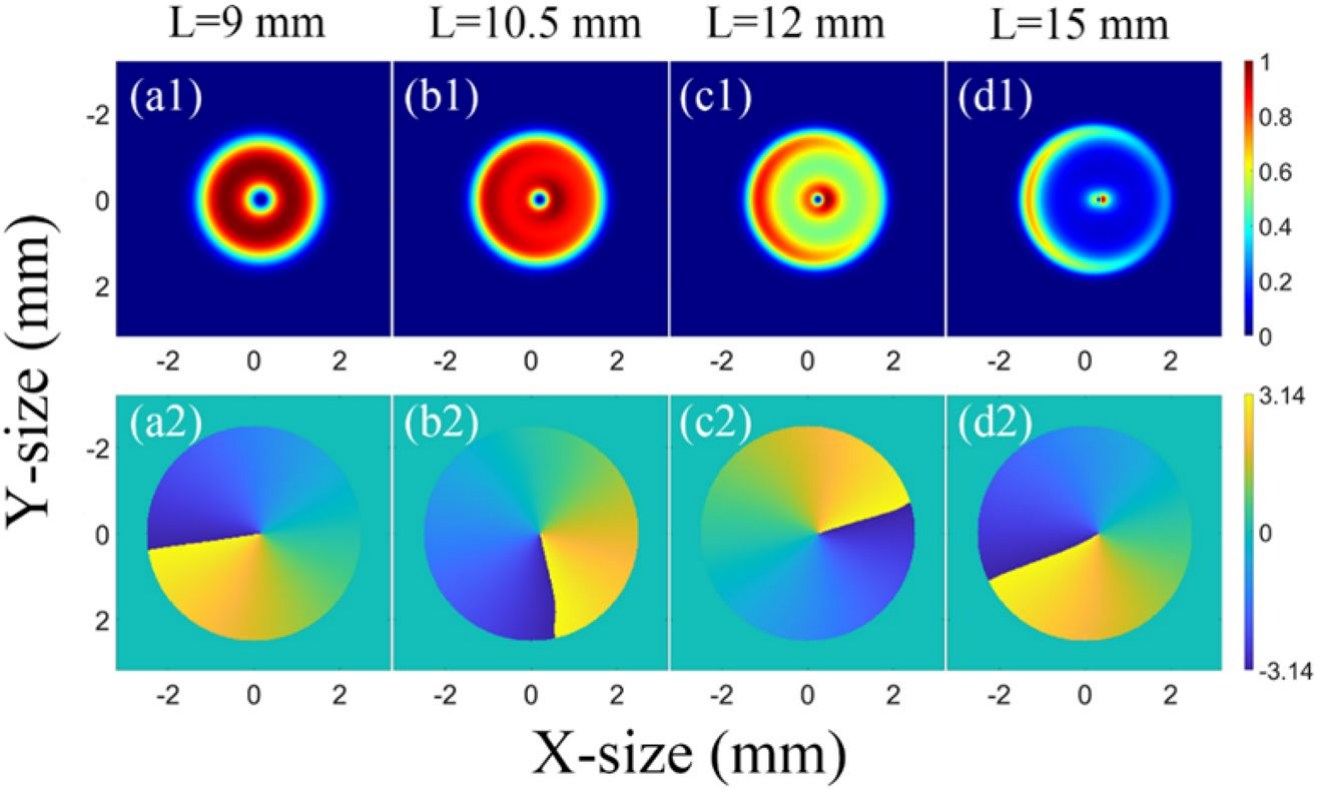
(**a**) Intensity distribution (**a1**–**d1**) and phase distribution (**a2**–**d2**) of amplified beams at different crystal lengths in non-collinear OPA.

In the OPA process, the obvious reason for the decrease of the proportion of the vortex beam with $l=1$ is that the near-field intensity and phase distribution change compared with the ideal LG01 beam. The intensity and phase distributions of the input ideal vortex beam with $l=1$ are illustrated in Fig. 5. The intensity and phase distributions of the amplified beam in NOPCPA at different crystal lengths of 9, 10.5, 12, and 15 mm are shown in Fig. 6. As depicted in Fig. 6, the amplified beams have distortions in both the intensity and phase distribution, especially after the saturation point with a loss in the axial symmetry. These distortions affected the specific intensity and phase distribution of vortex beam, and made the proportion of vortex beam of $l=1$ decrease and vortex beam of other TC increase.

**Figure 7:**
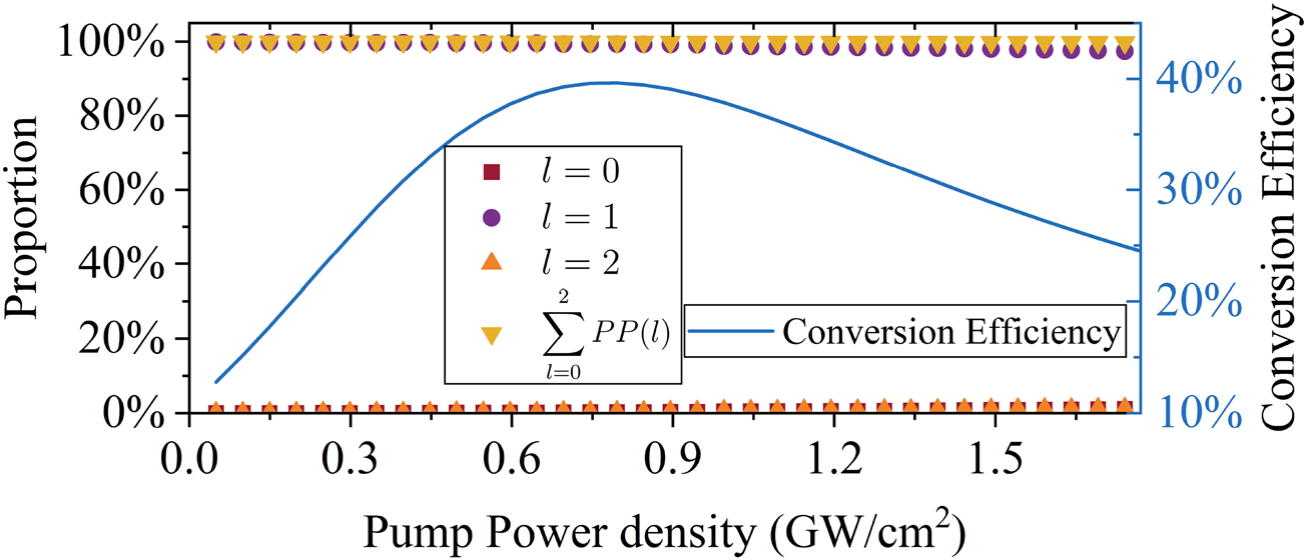
Change of OAM spectrum (left y-axis) and change of conversion efficiency (right y-axis) with different pump power densities.

As shown in Fig. 3, the proportion of the vortex beam with $l=1$ decreases with an increase in the crystal length. Here, the change of proportion of $l=1$ with an increase in the pump power intensity when the crystal length is fixed at 15 mm is illustrated in Fig. 7. The saturation point is at $I_{pump}=$ 0.80 GW/cm$^2$; it can be observed that the proportion of $l=1$ decreases by 0.68% from 0.05 to 0.80 GW/cm$^2$ and by 1.88% from 0.80 to 1.74 GW/cm$^2$, which indicates that the decrease after saturation (from 0.80 to 1.74 GW/cm$^2$) is more than that before saturation (from 0.05 to 0.80 GW/cm$^2$). This illustrates that saturation causes a significant decrease of the proportion of $l=1$.

**Figure 8:**
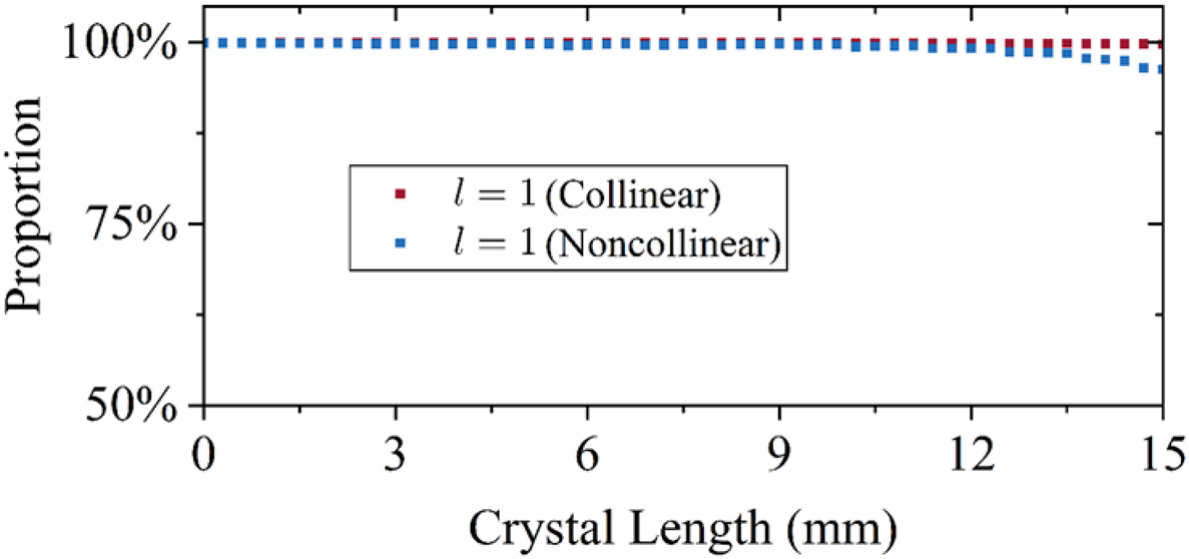
Proportion of $l=1$ of non-collinear and collinear OPA with crystal length (red and blue points, respectively) at a central wavelength (800 nm), respectively).

**Figure 9:**
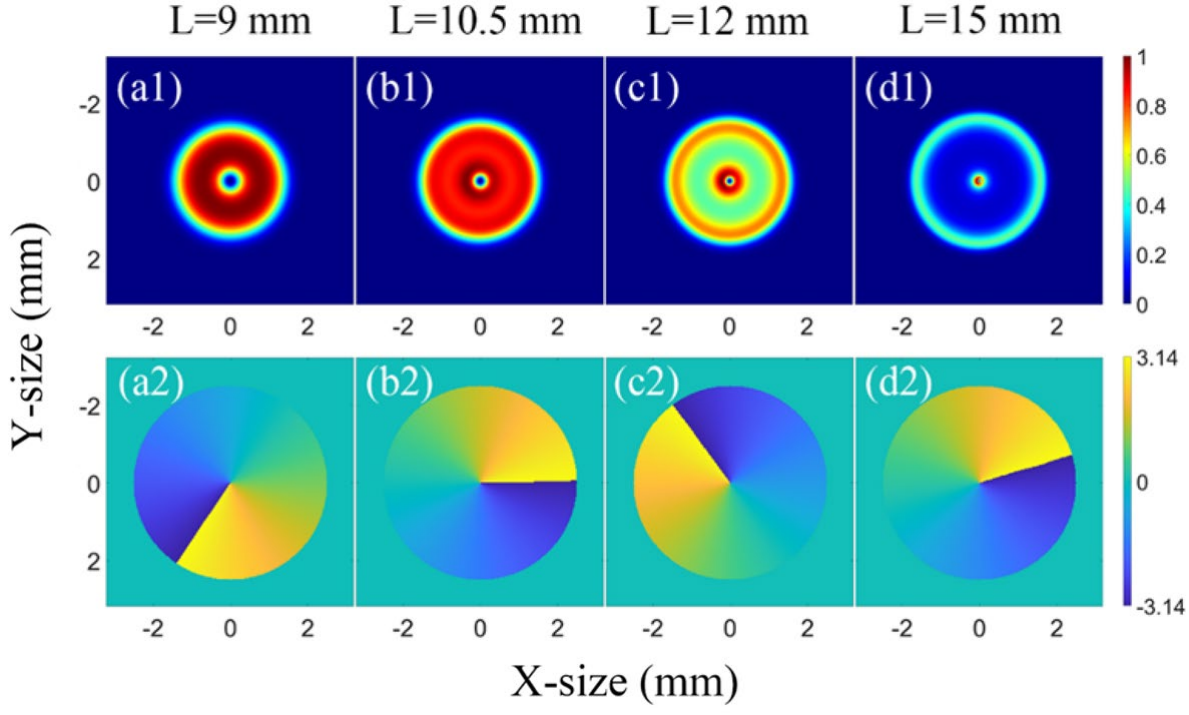
Intensity distributions (a1-d1) and phase distributions (a2-d2) of amplified beams ($l=1$ ) at different crystal lengths in collinear OPA.

**Figure 10:**
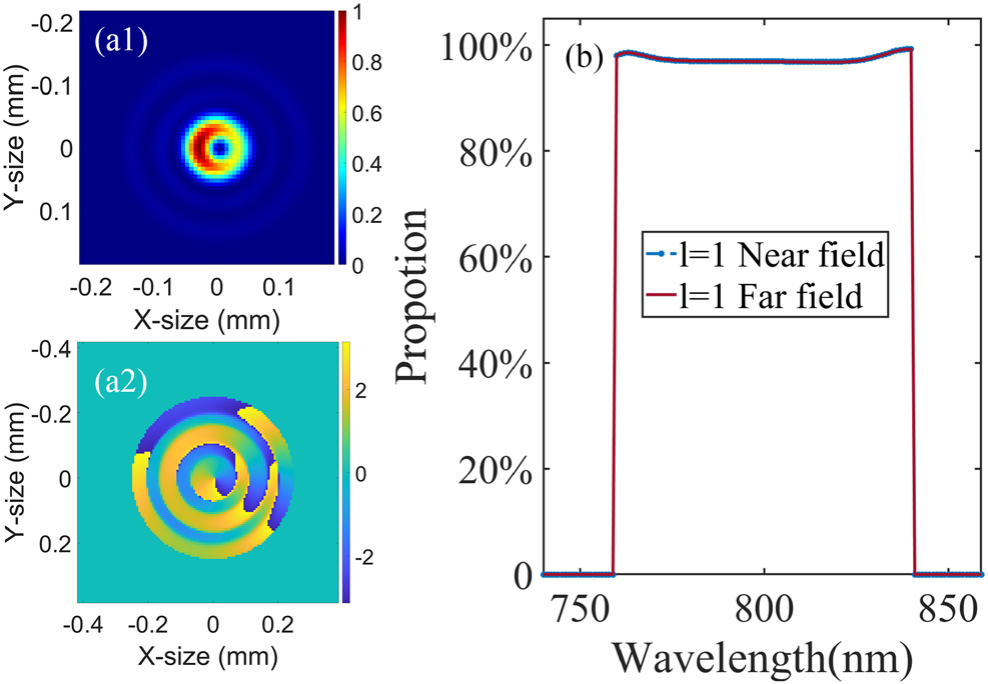
Focused intensity distribution (**a1**) and phase distribution (**a2**) of amplified beam corresponding to L = 15 mm. (**b**) Proportion of $l=1$ of the far field and near field with the wavelength.

### Collinear OPCPA structure

The above discussion is based on the non-collinear optical parametric amplification (NOPA) structure, the results of the collinear optical parametric amplification (COPA) structure are discussed in this section. For comparison, we calculate the 526.5 nm laser pumping 800 nm single wavelength laser in the non-collinear structure and 526.5 nm laser pumping 800 nm single wavelength laser in the collinear structure. The phase match angle of the COPA is $\theta =90^\circ$ and $\Phi =10.10^\circ$ for the LBO. The proportion curves of the 800 nm laser based on the NOPCPA are obtained from the broadband OPCPA shown in Fig. 3.

As depicted in Fig. 8, the proportion of $l=1$ in the collinear OPA only decreases by 0.21% from L = 0 to L = 15 mm, which is much less than the decrease in the NOPA (2.56%). The result illustrates that the non-collinear phase match is the most essential factor for the decrease of the proportion of $l=1$.

Fig. 9 shows the intensity and phase distribution of the amplified beam in the COPA. Compared with Fig. 6, the beam spots and phase distributions in the COPA have less distortions than the beam spots and phase distributions in the NOPA regardless of the crystal length, which is the reason that the COPA has less effects on the $l=1$ proportion of the LG beam than the NOPA.

### Far field characteristics

The far field spot and phase of amplified beam after being focused by an achromatic lens are depicted in Fig. 10a1 and a2, where the focal length is 200 mm. The near field and phase of the NOPA amplified laser beam are shown Fig. 6d1, d2. The far field spot has some distortions compared with the standard LG01 beam. In the annular distribution of far-field intensity, the left side is a little stronger than the right side, which is induced by the distortion of near field. The spiral phase in the far field still remains. The $l=1$ proportions of the far field are shown in Fig. 10b. The $l=1$ proportion of the far field does not have an obvious change compared with the near field. Therefore, the focus process does not have a distinct influence on the $l=1$ proportion regardless of the wavelength.

## Conclusion

In conclusion, we presented the numerically simulated results of the evolution in the proportion of the broadband Laguerre–Gaussian beam in LBO OPCPA. With a fixed pump intensity of 1.74 GW/cm^2^, the proportion of the vortex signal laser beam with 100% of $l=1$ decreased to 97.44% after amplification in a 15 mm length LBO OPA pumped by a 526.5 nm laser. The simulation results are consistent with our previous experimental results, which showed that the proportion of the vortex beam with a TC of 1 decreased from 72.87 to 67.51% in the OPCPA experiment. Our results shows that the non-collinear phase match is the most important factor for the decrease of the input topological charge ($l=1$) of the Laguerre–Gaussian beam. Meanwhile, for the amplification of the vortex by the OPA, the collinear OPA is better than the non-collinear OPA in maintaining the input topological charge. Besides, the saturation effect results in the decrease of the proportion of the central part wavelengths of the spectrum, which leads to the decrease of the $l=1$ proportion of the total OAM spectrum. The focus of amplified vortex beams is also calculated, which depicted that focus does not have an obvious influence on the $l=1$ proportion with the wavelength.

## Data Availability

The data generated and analyzed during the current study are available from the corresponding author on reasonable request.
